# Aeromedical Transport of Critically Ill Patients: A Literature Review

**DOI:** 10.7759/cureus.14889

**Published:** 2021-05-07

**Authors:** Alan Araiza, Melanie Duran, Salim Surani, Joseph Varon

**Affiliations:** 1 Critical Care, United Memorial Medical Center, Houston, USA; 2 Centro Universitario Médico Asistencial y de Investigación (CUMAI), Universidad Autónoma de Baja California, Tijuana, MEX; 3 Internal Medicine, Dorrington Medical Associates, Houston, USA; 4 Internal Medicine, Corpus Christi Medical Center, Corpus Christi, USA; 5 Internal Medicine, University of North Texas, Dallas, USA; 6 Critical Care, University of Texas Health Science Center at Houston, Houston, USA; 7 Critical Care, United General Hospital, Houston, USA

**Keywords:** aeromedical transport, aeromedical evacuation, critical care, emergency medical services, international transport, flight physiology

## Abstract

The aeromedical transport of critically ill patients has become an integral part of practicing medicine on a global scale. The development of reliable portable medical equipment allows physicians, emergency medical technicians, and nurses to transport wounded and diseased patients under constant critical care attention. Air transportation involves utilizing a fixed-wing (airplane) or rotor-wing (helicopter) aircraft to accomplish different types of transports ranging from scene responses to international transfers. The proper preparation and management of patients undergoing aeromedical transport require a basic understanding of the physiological changes and unique challenges encountered within the aircraft environment at 8,000 ft above sea level. The purpose of this paper is to review the literature and provide guidelines for approaching the aeromedical transportation of critically ill patients.

## Introduction and background

Aeromedical transport (AMT) represents the collaboration between aviation and medicine. The development of portable medical equipment, such as cardiac monitors, mechanical ventilators, infusion pumps, and suction pumps, among others, has permitted critical care units to extend beyond hospitals. At the present time, physicians, emergency medical technicians (EMT), and nurses can transport critically wounded or diseased patients via airplanes or helicopters, from a hospital, war zone, or catastrophe area to hospitals or tertiary care centers for definitive care [1-2].

Physicians of all specialties can decide to transport patients via air ambulance services. Despite this, there are no international guidelines that dictate the standard of care for preparation for an AMT and the expected patient care during the flight. Proper air transportation of critically ill patients requires an understanding of the physiological changes that occur at 8,000 ft above sea level within the aircraft environment [3].

**Figure 1:**
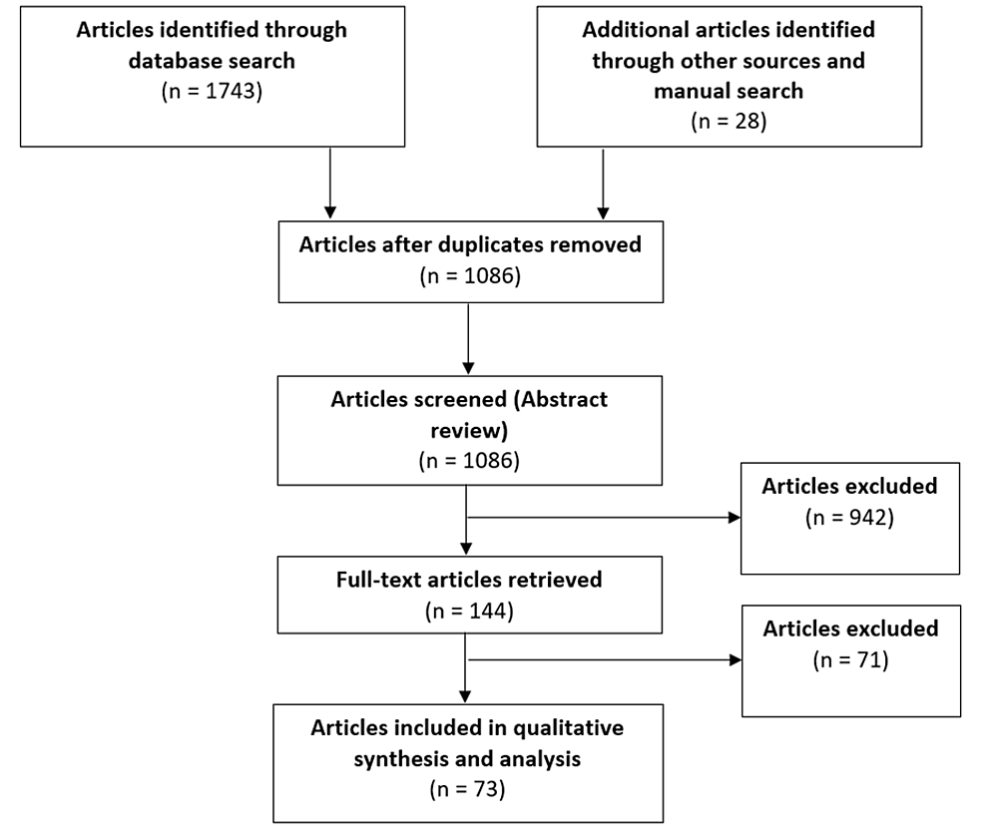
Flow chart of included articles

The purpose of this paper is to review all the relevant literature that studied the history of AMT, different types of aircraft utilized in transportation, medical personnel and equipment needed on board, epidemiology, physiological changes undergone during flight, potential problems the flight crew should anticipate, how to properly prepare the patient for a flight transfer, tasks the flight crew must carry out throughout the flight, and circumstances in which AMT is contraindicated. A multi-method approach was used to identify relevant studies for this review. Authors independently searched the National Library of Medicine’s Medline and Springer International Publishing databases for scholarly resources published from 1982 to 2020 using the following keywords and medical subject headings: aeromedical transport, aeromedical evacuation, international aeromedical transport, aeromedical transport epidemiology, aeromedical transport, and critical care (Figure 1). Bibliographies of all selected articles and scholarly resources that included relevant information on aeromedical transport were also included. This search strategy was done extensively until no new potential citations were found. Articles were excluded from our review if full-text was not retrievable or the subject was not within the scope of our review.

## Review

History

The origins of air medical transport date back to the Montgolfier brothers, who were known as the inventors of the hot air balloon in 1782 [1]. The first trial experiment the brothers conducted was on September 19, 1783, where they placed a duck, sheep, and rooster on board a hot air balloon to evaluate how altitude affected living beings. After eight minutes of flight that covered two miles and obtained an altitude of about 1,500 ft, the animals landed safely and were found unharmed. Then, one month later, the first known human flew in a hot air balloon [4].

In 1917, the Dorand AR II, a French aircraft, was the first air ambulance to transport a patient. Modern era AMT started during World War II, in which more than one million patients were transported just by the United States, with a mortality rate of 4/100,000 [1].

Starting in 1950, during the Korean war, rotor-wing aircraft, or helicopters, were created to get access to remote locations for medical transport due to rough terrain and unpaved roads in Korea [5]. Between 1965 and 1972, air ambulance helicopters rescued about one million civilians and injured soldiers in Vietnam [6-7]. The goal in using aircraft back then was to safely evacuate wounded soldiers from the battlefield and transport them to the care of doctors and nurses in a timely manner that could save their lives.

After the Vietnam War, however, the concept of AMT was no longer limited to military missions but also encompassed the civilian sector. Due to the success of AMT and continuous technological advancements, the number of air medical transports has continued to grow significantly in recent decades [8].

Types of air vehicles

Aeromedical transport can be divided into two main categories: fixed-wing (airplane) AMT and rotor-wing (helicopter) AMT. In general, ground medical care teams turn to fixed-wing or rotary-wing AMT when limitations of ground transportation interfere with patient care. Neither one is necessarily superior to the other, but there are specific characteristics that differentiate them (Table 1). The mode of transport should be chosen based on the different attributes that will benefit the individual’s patient care [9-10].

**Table 1:** Characteristics of fixed-wing and rotor-wing transport

Characteristics of fixed-wing and rotor-wing transport
Characteristics of Rotor-Wing Transport
*Speed:* Capable of maintaining speeds above 150 mph
*Vertical landing and takeoff:* Can access areas that are otherwise inaccessible for other types of vehicles
*Specialized medical equipment and personnel:* AMT services are based at tertiary care centers staffed with highly skilled personnel
Characteristics of Fixed-Wing Transport
*Speed:* Capable of maintaining speeds above 500 mph
*Specialized medical equipment and personnel:* Usually these airplanes are equipped with highly specialized medical equipment that satisfies the patient’s specific medical requirements

Attributes of Helicopter Transport

Speed: Rotor wing transport is capable of maintaining speeds of >150 mph and is generally used for distances shorter than 200 miles [11]. This, along with the ability to move from point to point, makes this mode of transport more useful in responding to acute events.

Accessibility: Vertical takeoff and landing allow for transporting patients in terrain that would be inaccessible for conventional ground ambulance vehicles like mountain climbing excursions, for example [9].

Specialized personnel and medical equipment: Aeromedical transport services are usually based in tertiary care centers that provide highly trained and skilled personnel [1].

Attributes of Airplane Transport

Speed: Airplanes can sustain speeds of >500 mph, which explains why they are used for distances greater than 200 miles, as they are more time and cost-efficient. They are not practical for distances under 200 miles due to restrictions in airport takeoff and landing [11].

Specialized personnel and medical equipment: Airplanes have highly trained and skilled personnel as well as specialized medical equipment. These are planned transports in which an AMT company can allocate equipment and personnel depending on the patient’s medical needs [10].

Types of aeromedical transports

These two categories of AMT vehicles are used to facilitate four main types of transport: scene response, transfers, specialty care, and evacuation. Although this is not an all-inclusive list, these groups cover the vast majority of flights [12].

Scene Response

The most common type of transport is the primary air transport, or scene response, in which an aircraft is considered the most efficient mode of transportation to save a patient in emergency scenes. This is mostly done by rotor-wing aircraft, and this transport is activated by ground crew taking into consideration the distance, time, and traffic patterns to definitive care before choosing to call for an air ambulance [12]. Emergency Medical Service (EMS) teams are obligated to opt for this alternative in circumstances where terrains are inaccessible to ground ambulances such as mountains, construction zones, and ocean rescues [9].

Transfers

Some community hospitals do not adequately meet the standards of treatment for medical conditions requiring specialized, advanced care. Therefore, a physician is sometimes faced with inter-hospital transfers for time-sensitive interventions such as patients with acute myocardial infarction, acute stroke, or severe trauma. In these cases, the patient’s prognosis could significantly benefit from minimizing arrival time to a cardiac catheterization lab, stroke center, or trauma center [9]. Under the Emergency Medical Treatment and Labor Act (EMTALA), the sending physician must stabilize and treat the patient, choose an optimum mode of transportation for that patient’s condition, and facilitate a smooth transition for the receiving physician [12].

Specialty Care

Medical conditions that involve advanced medical care and management during the flight, such as an intra-aortic balloon pump (IABP), extracorporeal membrane oxygenation (ECMO), and resuscitative endovascular balloon occlusion of the aorta (REBOA), require a well-trained and experienced medical crew who knows how to operate these specialized devices. It is more cost-efficient to assemble a specialty care team and use air transportation to quickly bring them to the hospitals or remote areas where they are needed rather than to equip each ground vehicle with these costly devices and transport the specialists [13-17].

Evacuation

When a physician determines that a patient requires a higher level of care than what the nearby health care facilities can offer, they can opt for a national or international aeromedical evacuation [18]. Patients who are traveling or living abroad may sometimes choose to continue their medical management in their home country. This is known as medical repatriation. Repatriation flights are common and can occur in travelers with unexpected emergencies or when a traveler develops a condition that requires higher-level care than is offered in the country they are visiting. Fixed-wing AMT is the optimal choice for critically ill patients who require special care and constant monitoring. The majority of repatriation flights do not require a physician on board, but some countries make it a requirement [12]. The rules and stipulations on international medical transportation vary depending on the departing and receiving jurisdictions [2,19].

Medical personnel and equipment on board

Currently, there are no Federal or International Standardized Guidelines for AMT. In addition, there are no guidelines that specify the qualifications a transport physician must have [20]. Aside from the pilot, the flight staff can include a combination of a physician, nurse, respiratory therapist, and/or EMT. The United States Air Force designed their own critical care air transport team (CCATT) for soldiers who suffer traumatic injuries. The CCATT team includes a critical care doctor, critical care nurse, and respiratory therapist [3]. Usually, in civilian air transport, critically ill patients are accompanied by a physician, nurse, and EMT. The physician must be trained to handle severe illness, potential in-flight patient deterioration, and any other in-flight adversities [8].

Critically ill patients who require a higher level of care in a non-emergency setting are usually not transferred in the acute phase of their disease but, rather, these transfers are planned, long-distance flights that require special considerations. The medical equipment on board is tailored to each individual patient’s needs, as there is no defined standard as to what equipment must be on board. A list of the basic recommended equipment for fixed-wing aeromedical transports is provided in Table 2 [2,21-22]. Unexpected weather changes, delays, or sudden deterioration of the patient’s condition could quickly deplete medical supplies. The flight crew must prepare for calamity and have a backup reserve of power supplies, oxygen tanks, monitors, and, especially, condition-specific medical equipment when transporting critically ill and/or injured patients [23].

**Table 2:** Recommended basic equipment for aeromedical transport

List of recommended basic equipment for aeromedical transport
Pulse oximeter
Electrocardiogram equipment or portable cardiac monitors
Blood-pressure monitors
Capnography equipment
Thermometer
Portable oxygen with a regulator
Backup oxygen tanks
Mechanical ventilator
Suction device with catheters and drainage-collection units
Intubation equipment
Tracheostomy kit
Nebulizers with necessary nebulization treatments
Nasal cannula
Nasogastric tubes
Intravenous needles and necessary tubing
Infusion device
Electrical converters for use of aircraft power source
Defibrillator with pads
Emergency resuscitation kit (including bag valve mask)
Medications for resuscitation
Medications tailored to patient’s needs
Gloves
Pair of tape scissors
Surgical kit
Bandages and wound dressings
Wound treatment kit
Doppler monitor
Backup battery packs
Earplugs for the patient (Rotor-wing)
Cervical spine collars

Epidemiology

Even though thousands of international aeromedical evacuations occur annually, very few epidemiological studies exist. Coste et al. analyzed data for all the medical evacuations (MEDEVAC) carried out by the French Forces from 2000-2010. During this time period, 420 MEDEVACs were carried out, transporting 529 patients, with 90% of these being French soldiers. The most common original location of evacuation was Europe representing 42%, followed by 39% from Africa, and 17% from the Middle East. Trauma occurred in 49.5% of patients; 30.2% of patients had other medical conditions, 10.2% of transports were with nontraumatic surgical patients, 6.8% were burn patients, and 2% were psychiatric patients [24].

In 2010, Sand et al. performed a retrospective chart review of 504 aeromedical repatriation evacuation cases, which were classified and analyzed according to the specialty in charge prior to the flight transfer. According to this study, the most frequent specialty cases requiring aeromedical evacuation were trauma surgery, internal medicine, and neurology. The most frequent diagnoses were patients with femoral neck fractures, cerebrovascular accidents, and myocardial infarctions [19].

In 2019, Kim et al. performed a retrospective descriptive analysis on 33 Korean patients who were injured while traveling from 2013 to 2017 and repatriated to South Korea. Trauma occurred in 28 patients, with pedestrian injuries being the most common. Of the other five non-trauma cases, acute myocardial infarction was most common. The most common diagnosis among these patients was brain injury in 19 cases, followed by fractures in 10 cases, and the remaining four cases were categorized as “other” diagnosis [25].

Aviation physiology

In order for healthcare providers to take part in an AMT, aviation physiology must be fully understood. The physiological changes that occur in the flight environment should be taken into consideration when interpreting the clinical vignette of the patient if any changes occur. For this reason, it is essential to understand the chemical composition of the atmosphere, which is a mixture of numerous gases, composed mainly of nitrogen (78%) and oxygen (21%). The sum of the weight, or forces, of all the gases that comprise the atmosphere, is equal to the atmospheric pressure, which at sea level is measured as 760 mmHg [26]. The composition of air remains constant at any altitude, but the atmospheric barometric pressure will decrease exponentially as altitude increases due to the kinetic properties of gases as explained by the physical gas laws [27].

Dalton’s law states that the total pressure of a mixture of gases is equal to the sum of the partial pressure of each gas in the mixture:

P_total_ = P_1_ + P_2_ + P_3_ + …

Again, atmospheric pressure is the total sum of all the partial pressures of gases that comprise it [28]. This explains why as ambient altitude increases, the atmospheric pressure drops due to a decrease in the partial pressure of each gas. At 8,000 ft, oxygen still represents 21% of the atmosphere’s composition, but oxygen’s partial pressure (PO_2_) is 108 mmHg as compared to 148 mmHg at sea level, leading to arterial desaturation [29].

Boyle’s gas law states that when the temperature remains constant, as it happens in the human body, a volume of gas is inversely proportional to its pressure:

P_1_ * V_1_ = P_2_ * V_2_

As the pressure drops, Boyle’s law predicts the gas expansion of gas-filled body cavities and medical devices, like endotracheal tubes (ETTs) cuffs, intravenous (IV) fluid bags, etc. [30]. During airplane flights, gas can expand up to 35% from its baseline at sea level. It is imperative that healthcare providers keep this gas law in mind throughout the entire transport [2].

Henry’s law states that the amount of gas in a solution varies directly with the partial pressure that the gas exerts on the solution:

C = k * P

Therefore, if the pressure of the gas over the solution decreases, the gas will escape the solution [22]. This is the physical explanation for why carbon dioxide bubbles are released when carbonated beverages are opened and why nitrogen bubbles may come out of body tissues’ solutions, which can lead to altitude-induced decompression sickness [3].

Aircraft ambiance

Most civilian commercial flights cruise at altitudes between 25,000 and 45,000 ft [30]. Under normal conditions, humans can tolerate flying at altitudes of up to 10,000 ft without supplemental oxygen therapy. In order to counteract the detrimental effects that aviation physiology has, all civilian flights are subject to standardized pressurization dictated by the cabin altitude. This aids in maintaining a normal partial pressure of oxygen (PO_2_) and partial pressure of arterial oxygen (PaO_2_) during flights [27,30].

The term pressurization refers to an increase of pressure inside the aircraft when compared to the outside. Pressurization allows humans to fly at altitudes of up to 50,000 ft above sea level without experiencing major clinical symptomatology [31]. Cabin altitude sets the effective altitude, thus pressure, to which passengers are exposed, thereby limiting the pressure fluctuations. For example, if a plane is flying at an altitude of 50,000 ft above sea level, the pressure inside the cabin is kept between 6,000 and 8,000 ft above sea level. The Federal Aviation Administration (FAA) requires all civilian flights (fixed-wing) to be pressurized at cabin altitudes of no more than 8,000 ft at maximum operating altitude. Due to this reason, cabin altitude is kept between 6,000 and 8,000 ft above sea level, rather than being pressurized to sea level. The air inside the cabin is compressed, allowing a life-compatible PO_2_ [26,32].

Most aircrafts used in civilian AMT were not originally designed to transport patients and are usually aircraft adapted for medical evacuations [33]. Therefore, the physical stressors derived from the aircraft environment will affect not only the patient but also the medical staff and equipment. The classic flight stressors include gravity force and changes in altitude, temperature, humidity, acceleration, noise, and vibrations. These physical stressors, excluding noise and vibration, are greater in fixed-wing transports as these vehicles are flown at a higher altitude [1,10].

Physiological translation

**Figure 2:**
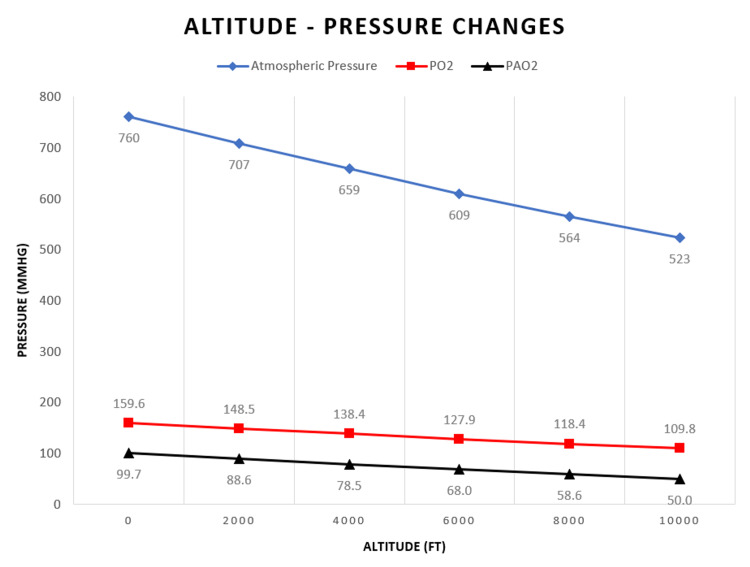
Relationship between altitude and atmospheric pressure PO_2_ = Partial Pressure of Oxygen; PAO_2_ = Partial Pressure of Alveolar Oxygen

The physiological translation of physical gas laws, aircraft environment, and physical stressors raises a distinct clinical scenario for patients and the flight team. The primary physiological difference between air and ground environment is altitude. As the aircraft ascends, the atmospheric barometric pressure drops due to a reduction in the partial pressure of the gases that comprise it, including PO_2_ [20]. The inverse relationship between altitude and atmospheric pressure is depicted in Figure 2.

At sea level, the atmospheric pressure equals 760 mmHg and PO_2_ is 160 mmHg or 21% of the atmosphere’s pressure [34]. However, this PO_2_ is not equivalent to the oxygen available for diffusion within the alveoli. As air is inhaled by the upper airway, it is warmed and humidified, which adjusts the alveolar partial pressure of oxygen (PAO_2_), as explained by the alveolar gas equation [35]:

PAO_2_ = (FiO_2_ * (P_Atm_ - PH_2_O)) - (PaCO_2_ / RQ)

Where PAO_2_ is the partial pressure of oxygen in the alveoli; P_Atm_ is the atmospheric pressure which decreases as altitude increases; PH_2_O is the partial pressure of water, which usually is 47 mmHg; PaCO_2_ is the partial pressure of carbon dioxide, which under normal conditions is between 35-45 mmHg; the FiO_2_ is the fraction of oxygen, which remains constant at 21% for the first 15 miles of the atmosphere [34]. Under physiological conditions, the PAO_2_ equals:

PAO_2_ = (0.21 * (760 - 47)) - (40/0.8) = 99.7 mmHg

In the lungs, specifically the alveoli, oxygen needs to diffuse across the alveolar-capillary barrier in order to reach the arterial circulation, which eventually reaches all the tissues of the body. Under normal conditions at sea level, the PaO_2_ should be between 75 and 100 mmHg, or almost equal to the PAO_2_ value. These PaO_2_ values are high enough to satisfy the metabolic demands [34-35]. As altitude increases during air medical transports, the PaO_2_ is inversely reduced (Table 3) [31].

**Table 3:** Relationship between altitude and partial pressure of oxygen PO_2_ = Partial Pressure of Oxygen; PiO_2_ = Partial Pressure of Inspired Oxygen; PAO_2_ = Partial Pressure of Alveolar Oxygen; FiO_2_ = Fraction of Inspired Oxygen

Relationship between altitude, atmospheric pressure, and oxygen pressures						
Altitude (ft)	Altitude (m)	Atmospheric pressure (mmHg)	PO_2_ (mmHg)	PiO_2_ (mmHg)	PAO_2_ (mmHg)	Equivalent FiO2 at sea level (%)
0	0	760	159.6	149.7	99.7	21.0
2000	610	707	148.5	138.6	88.6	19.4
4000	1220	659	138.4	128.5	78.5	18.0
6000	1830	609	127.9	118.0	68.0	16.6
8000	2440	564	118.4	108.6	58.6	15.1
10000	3050	523	109.8	100.0	50.0	14.0
12000	3660	483	101.4	91.6	41.6	12.8
14000	4270	446	93.7	83.8	33.8	11.8
16000	4880	412	86.5	76.7	26.7	10.8
18000	5490	379	79.6	69.7	19.7	9.8
20000	6100	349	73.3	63.4	13.4	8.9
22000	6710	321	67.4	57.5	7.5	8.1
24000	7320	294	61.7	51.9	1.9	7.3
26000	7930	270	56.7	46.8	0.0	6.6
28000	8540	247	51.9	42.0	0.0	5.9
30000	9150	226	47.5	37.6	0.0	5.3
35000	10700	178	37.4	27.5	0.0	3.9
40000	12200	141	29.6	19.7	0.0	2.8
45000	13700	111	23.3	13.4	0.0	1.9
50000	15300	87	18.3	8.4	0.0	1.2

Contrary to common belief, cabin altitude does not eliminate the risk for side effects related to altitude [36]. Hypoxemia is the major threat encountered during AMT due to hypobaric hypoxia, a decrease in atmospheric pressure [26,29,37]. At 8,000 ft, the atmospheric pressure is 565 mmHg, translating to a PAO_2_ of 53-64 mmHg (normally 99.7 mmHg), and oxygen saturation (SpO2) between 85 and 91% [31]. In other words, breathing air at 8,000 ft above sea level is the same as breathing air with an FiO2 of 15.1% at sea level [29].

**Figure 3:**
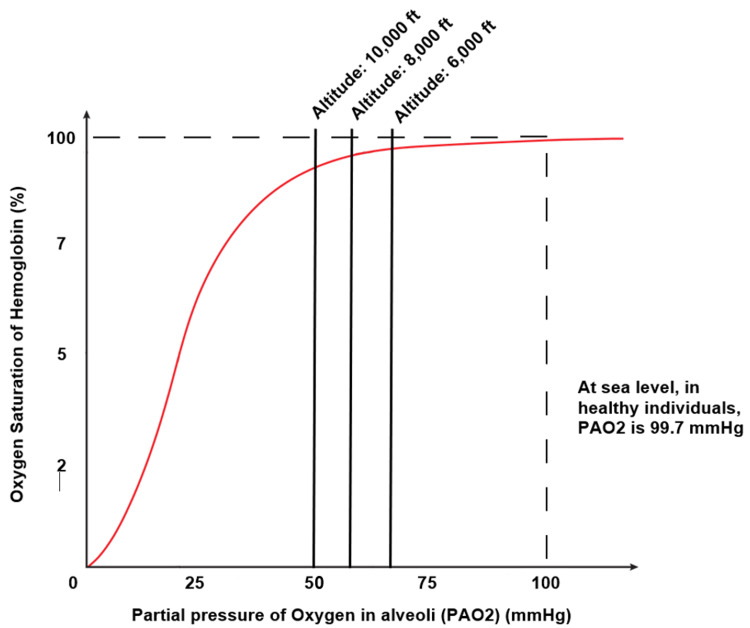
Oxyhemoglobin curve at altitude

In healthy people, this would not raise any relevant symptomatology. However, in patients who have underlying medical conditions that affect their baseline (at sea level) PaO_2_ values, the altitude-induced reduction of PaO_2_ can be dangerous [26]. Decreasing the PaO_2_ past the 53-mmHg mark places patients in the steeper, and more dangerous, section of the oxyhemoglobin curve (Figure 3), which means a lower affinity of hemoglobin for oxygen. Small reductions in PaO2 in this section of the curve can produce a significant decrease in SpO2 [22,38].

Hypoxia is easily identifiable with continuous pulse oximetry and can be corrected via supplemental oxygen therapy [39-40], but gas expansion can be more difficult to recognize mid-flight [2]. For cost-efficiency, cabin altitude is kept between 6,000 and 8,000 ft in both commercial and private aeromedical transfers. This means that gases expand about 35% from their original volume. Gas expansion explains most clinical presentations of barotrauma during flights: aerotitis, aerosinusitis, aerodontalgia, and, of special concern during AMT, abdominal barotrauma and expansion of pneumothorax [1,36].

Medical equipment is also affected by gas expansion, as well as other phenomena related to the physical stressors derived from the aircraft environment. Changes in barometric pressure, with consequent changes in gas volume, are of special interest in blood pressure cuffs, intravenous (IV) fluid bags, and ETT cuffs [1,22].

During ascent and descent, the gas volume will change, either expanding or contracting, respectively. These changes in gas volume can affect non-invasive blood pressure (NIBP) measurements, providing momentary false readings. Intravenous fluid solution bags have air within them, which can alter the drip rate from the original desired rate as gas volume changes. For this reason, IV fluid bags should be used in conjunction with infusion pumps, and not by mere drip rate [41]. The ETT’s cuff air, thus pressure, will increase during ascent and will decrease during descent. At 8,000 ft, ETTs cuff pressure triples (almost quadruples) from baseline at sea-level (25 mmHg vs. 90 mmHg) [3]. During descent, the cuff pressure decreases, which can allow leakage around the cuff and can cause aspiration pneumonia [42]. For this reason, filling up the ETT cuff with normal saline has been shown to maintain a steady cuff pressure throughout the flight at standard cabin altitudes [43-44].

Vibrations are more noticeable and play a bigger role in rotor-wing than in fixed-wing flights. Nevertheless, vibrations should be considered when interpreting pulse oximeters and cardiac monitor measurements [41,45].

By considering the physiological changes that occur not only in the patient but also in the medical flight team and the medical devices, preparation for the transport can be done appropriately.

Patient examination and preparation for aeromedical transport

The type of AMT determines the possibility of meticulously preparing and tailoring the transfer according to the specific patient’s needs. Perfect and ideal planning might not be possible in scene responses or emergency evacuations, which is why AMT teams for these transfer types usually have standard medical equipment and medicines. On the other hand, long-distance AMT via fixed-wing aircraft are usually planned transfers. Careful assessment and preparation for the long-distance AMT of critically ill patients cannot be overstated [41].

Nowadays, it is common in planned AMTs for the departing hospital to fax the patient’s medical records to the AMT company. By doing this, the company’s medical director can assemble an appropriate flight team, usually a combination of EMT, nurse, and, for critical patients, a physician [8]. All the members of the flight team are responsible for carefully reading the patient’s files and understanding the patient’s medical condition. If possible, the flight team should visit and examine the patient in the hospital days prior to the transfer. This allows the team to better understand the patient’s condition, enhancing the preparation and planning for potential complications throughout the course of the transfer [41].

Regardless of the flight team composition, all members must make an effort to be present at the departing hospital at the time of the patient transport. It is imperative to talk to the physician in charge of the patient, as well as the nursing staff [1,41].

By systematically examining the patient, the flight crew can evaluate if the patient has been stabilized appropriately prior to the AMT. If further interventions are required, all of them must be performed in the departing hospital, in a controlled environment [2]. The required medical equipment for the appropriate continuation of care should be ready. Backup equipment should be available during the transport. Electrical equipment needs to be compatible with the aircraft’s electrical circuits and enough batteries should be on board.

The main goal of a flight team transporting a critically ill patient is to ensure the smoothest transition between facilities. Careful examination of the patient is probably the most crucial aspect for adequate preparation for the flight. We recommend a system-oriented approach for critically ill patients [46-47].

Neurological

Understanding the current neurological status of the patient can be challenging, as the flight crew usually has not examined the patient prior to the transport. What was the patient’s baseline status? Have there been seizures? Any anxiety disorders? These are all basic questions that must be asked. Neurological evaluation should at least include a Glasgow Coma Scale (GCS), cranial nerve examination, muscle strength, and deep tendon reflexes [20,41].

Special consideration should be given to sedated patients. A sedation strategy should be planned out. Flight crews should consider the amount of sedation the patient has required and how it worked. A recent study reported that bispectral index (BIS) monitoring during AMT is reliable in sedated or anesthetized patients. Keeping the patient calm during the transport is a priority [48-49].

Cardiovascular

Patients being aero-medically transported must be hemodynamically monitored throughout the whole transfer. Focus should be given to the heart rhythm and rate, as well as blood pressure [45,50].

Dysrhythmias are a common finding in patients with heart failure and cardiomyopathy. Patients who are considered at risk of presenting dysrhythmic episodes require a therapeutic strategy on board (medications, defibrillator, etc.) [20].

The patient’s blood pressure trend can help assess the hemodynamic status and need for continuous invasive monitoring. If deemed hemodynamically unstable, patients should be monitored through invasive (intra-arterial) blood pressure (IBP), rather than NIBP monitoring. McMahon and colleagues compared NIPB with IBP during AMT and concluded that for patients requiring accurate blood pressure measurements during AMT, IBP remained the gold standard. When NIBP is the only option, mean arterial pressure (MAP) is more reliable than systolic blood pressure alone [51].

Pulmonary

Making sure the patient has a secure airway throughout the transfer is the main priority when assessing the pulmonary system. If the patient has any risk of losing airway patency, even if low, intubation prior to transportation is appropriate [2]. Patients under mechanical ventilation require a lung-protective strategy [33].

Deciding the optimal mechanical ventilation mode is often controversial and is usually based on the flight crew's preference. The most common modes include: assist control (AC), synchronized intermittent mandatory ventilation (SIMV), and pressure support ventilation (PSV). Regardless, no mode of ventilation has shown to improve outcomes when compared to another one. Another controversial discussion is the use of pressure-controlled vs. volume-controlled ventilation. Neither of them impacts the outcome, and its use should be guided by preference and experience [52].

Regardless of the selected mechanical ventilation mode, a lung-protective strategy should be elected during AMT. Initial tidal volume (V_T_) should be 4-8 mL/kg of the predicted body weight (PBW), as lung volumes are mainly determined by height and not body weight [52-53].

PBW men = 50 + 2.3 (height in inches - 60)

PBW women = 45.5 + 2.3 (height in inches - 60)

Despite utilizing a V_T_ based on PBW, there is no well-defined “safe” V_T_, but maintenance of plateau pressure (Pplat) below 30 cm H2O has shown to improve mortality outcomes.

Positive end-expiratory pressure (PEEP) is used to maintain end-expiratory lung volume to improve lung recruitment and oxygenation. A PEEP of 5 cm H_2_O is considered physiologic and should be used in all mechanically ventilated patients. The level of PEEP should be balanced appropriately to improve alveolar recruitment without causing lung stress or atelectrauma by overdistension [54]. A respiratory rate (RR) between 12 and 15 per minute is standard. The goal is to maintain minute ventilation of approximately 10 L/min. Patients with chronic obstructive pulmonary disease with a risk of hyperinflation should have an inspiratory/expiratory (I/E) ratio of 1:2 to 1:3, in order to prevent air trapping and auto-PEEP [52].

These days, portable ventilators have a similar performance to those seen in intensive care units (ICU). Ventilators used in AMT should be certified as airworthy by aeronautical agencies (e.g. the US Federal Aviation Administration). Transport ventilators need to be able to compensate for altitude changes, deliver consistent V_T_ and minute ventilation, as well as provide accurate volume monitoring [55-56].

For any transport in which the patient requires supplemental oxygen therapy, the estimated oxygen requirements should be calculated [57]. A simplified formula for calculating estimated oxygen liters is:

Oxygen needed (L) = (Minute volume) * (FiO_2_) * (Time)

Minute volume (L/min) = ((V_T_) * (Ventilator respiratory rate per minute)) / 1000

Where the estimated oxygen needed will be in liters (L), minute volume is expressed in L/min, FiO_2_ in a range of 0 to 1 (e.g. 50% is 0.5), time in minutes (estimated number of minutes of the transfer), V_T_ is expressed in milliliters (mL), and respiratory rate is per minute [58].

Due to the low humidity experienced onboard during AMT, a heat and moisture exchanger is highly recommended for transports. The selected device should have a low dead space (< 50 mL) to allow lung-protective V_T_ [41,52].

Endotracheal tubes serve as a passage for ventilation while protecting the lower airways from aspiration. Traditionally, ETTs are insufflated with air, which can expand or deflate during ascent or descent, respectively, due to the barometric pressure changes. The use of normal saline to fill up the ETT cuff has shown to maintain a steadier cuff pressure throughout the flight, but pressure at sea level exceeds the threshold associated with tracheal mucosal blood flow occlusion. For this reason, if normal saline is to be used, the ETT cuff should be filled with air while measuring the volume, then the air should be removed and the equivalent volume of normal saline should be used to fill the cuff. The usage of saline must be weighed against the fact that most ETT manufacturers advise against using saline insufflation [44,59]. A more recent study showed a benefit from utilizing an automated ETT cuff pressure device at altitude [42].

Prior to transporting the patient out of the departing hospital, a pneumothorax should be ruled out by a same-day chest X-ray. Another goal is to have a patient’s baseline SpO_2_ of > 94% at sea level as the hypoxic environment at altitude will drop this SpO_2_ [41,52].

Renal and Metabolic

Fluid inputs and outputs (I/Os) of all critically ill patients need to be reviewed prior to AMT. Volume overload can be challenging to recognize mid-flight due to the lack of diagnostic equipment. If not done already, a Foley catheter should be placed prior to transport. This will allow strict I/O monitoring. Patients with any type of kidney disease (acute, chronic, or end-stage) should be specifically monitored for urine production throughout the transfer [1,60-61].

Experienced hospitals with AMT will order a metabolic panel prior to transfer and will correct electrolyte imbalances if required. The flight crew should request this to be done prior to the transfer, as any electrolyte imbalance may be difficult to identify during the flight without any laboratory tools available. Patients who regularly get hemodialysis should do so on the same day pre-transport [10,41].

Gastrointestinal and Nutritional

As in other enclosed gas spaces, the gastric bubble should be decompressed prior to air transport. If not done already, an orogastric (OG) or nasogastric (NG) tube should be placed with low intermittent suction. This will contribute to patient comfort at altitudes of 8,000 ft above sea level. At the same time, these tubes allow the administration of medications and enteral feeding, which is especially relevant in intercontinental flights. For appropriate caloric feeding, basal energy expenditure with the Harris-Benedict caloric intake adjustment should be calculated [1-2].

Some hospitals place a rectal tube specifically for transport [41]. This facilitates the flight crew's job throughout the transfer, but it is not a requirement for AMT.

Skin and Extremities

When transporting a critically ill patient for AMT, the patient should be examined for any wounds or pressure ulcers. If possible, the departing hospital staff should assist with wound care (WC) prior to the transfer and should instruct the flight crew on how and when to continue the WC. This aspect is often neglected during AMT, but when transporting patients who require WC, there should be supplies on board [62-63].

Infectious Diseases

Most patients with infections can be transported without complications, avoiding cross-contamination between patient and medical staff, by following basic hygiene measures like handwashing and protective equipment usage. Known “common” infections require a batch of antibiotics that will last the estimated time of the transport. Hospitals usually provide the flight crew with antibiotics, but if not, accommodations should be made to have them on board. Untreated infections can develop into sepsis syndrome which can be fatal, especially at 8,000 ft above sea level [64].

Patients with highly contagious infections can be transported in isolation chambers, accompanied by specialty care teams. Despite the complexity of these transfers, there are no defined guidelines that tell health care professionals how to prepare for these flights [65-66].

Hematological

The two main hematological concerns are the hemoglobin level and the preferred deep vein thrombosis (DVT) prophylaxis strategy. A same-day complete blood count should be provided by the departing hospital. Patients with hemoglobin values (< 7.0 g/dL) should be transfused prior to the transfer [67]. Deep vein thrombosis prophylaxis should be continued, as the use of sequential compression devices (SCD) would require a recent venous ultrasound of bilateral lower extremities, as well the SCDs being certified as airworthy [36].

By examining all of the above, medical care can be provided appropriately during the transfer.

Flight tasks

Nowadays, air ambulances can be nearly as sophisticated as ground-based ICUs, which allows for the continuation of almost all medical and nursing tasks during flight. Patient care should be continued as similarly as possible prior to the AMT [3,41].

Critically ill patients are monitored at all times in ground-based ICUs, and it should be no different in air-based ICUs. From the moment of picking up the patient until the definitive delivery, the patient must be at least in constant cardiac, pulse oximetry, and blood pressure (NIBP or IBP) monitoring [39-40,45,51]. Other parameters that should be monitored, if appropriate, are end-tidal carbon dioxide (ETCO_2_), BIS, or intracranial pressure [48,68-69]. Constant monitoring of the patient allows for the prompt detection of possible problems (e.g. hypoxia, dysrhythmias, hypotension, etc.).

Hospital medications should be continued as scheduled, guided by the updated list of medications requested prior to the transfer. A useful thing to do on long flights is to set up timers for the scheduled medications. Keeping track of the medications can sometimes be difficult, especially without an electronic medical record and with multiple changes in time zones during the flight [41,62].

Patient comfort is a priority, as the flight environment will be uncomfortable and stressful for patients. The basic considerations to keep in mind are providing pain medication throughout the transfer, measuring and controlling temperature throughout the flight, utilizing eye lubrication to prevent corneal ulcers, and repositioning the patient every two to three hours to prevent pressure ulcer development [70-72].

As mentioned, the main job of the flight crew is to provide the smoothest possible transition between hospitals. In order to do so, from our experience, we recommend writing a medical flight note, not only for legal documentation but also for the receiving hospital. Ideally, this note should be written similarly to an in-patient progress note, in a system-oriented manner. The note should encompass everything done from patient pick until delivery, and if possible, the complete history of present illness based on the medical records obtained from the departing hospital. By doing this, the receiving hospital will have an easy-to-read note that encompasses, in a systematic way, the patient’s medical history.

Contraindications

When assessing whether or not to send a patient on an AMT, risks and contraindications need to be considered above all. Unsafe weather conditions, determined by the pilot, are an absolute contraindication. In addition, if possible, the patient should be hemodynamically stabilized before undergoing a flight mission. Lastly, flying an uncontrollable or combative patient may create an unsafe environment for the aircraft and crew. Obtaining the proper level of sedation, placing restraints, or having a family member on the flight may appease the situation [12]. In the case of trauma patients, fractures should be reduced and spinal injuries should be immobilized.

The relative contraindications in the setting of rotor-wing aircraft are related to spatial limitations such as pregnancy with impending delivery or cardiopulmonary arrest. Both of these scenarios require a flight crew member to be without a seatbelt for a prolonged period of time to either deliver the baby or to perform lifesaving cardiopulmonary resuscitation (CPR) [2].

Relative contraindications encountered mainly, but not exclusively, on fixed-wing transports are patients with medical conditions that may be negatively impacted by the higher altitude changes maintained on fixed-wing transport. These include hemorrhagic cerebrovascular accident (CVA), pneumocephalus, barotrauma, pneumothorax, pneumoperitoneum, and any type of recent surgery within a seven-day time frame [2,73]. As no written regulations exist regarding the optimal postsurgical waiting time before sending a patient on a flight transport, this should be a joint decision made by the treating and receiving physicians, along with the flight crew.

## Conclusions

It is undeniable that the different types of AMT now form an integral part of medicine. Daily, medical staff around the world come across patients with an acute condition that requires emergent transportation to a more specialized facility. For this reason, it is imperative to have a good understanding of flight physiology and comprehend the potential risks the patient may be subject to in order to properly determine when the benefits outweigh the risks. These emergency air medical systems make it possible to provide specialized care for the critically injured and ill.
